# PLGA-coated methylene blue nanoparticles for photoacoustic imaging and photodynamic/photothermal cascaded precisely synergistic therapy of tumor†

**DOI:** 10.1039/d1ra07689b

**Published:** 2022-01-10

**Authors:** Xiaomu Xu, Haiyan Wu, Yue Yang, Bo Liu, Jijing Tian, Huihui Bao, Tianlong Liu

**Affiliations:** College of Veterinary Medicine, China Agricultural University No. 2 West Road Yuanmingyuan Beijing 100193 P. R. China; College of Chemistry and Materials Science, Jinan University No. 601, West Huangpu Avenue Guangzhou Guangdong 510632 China; China National Center for Food Safety Risk Assessment No. 37, Guangqu Road, Chaoyang District Beijing 100022 P. R. China

## Abstract

Photodynamic therapy (PDT) and photothermal therapy (PTT) are synergetic treatment strategies in antitumor treatment to achieve the best anticancer efficacy. Although traditional photosensitizer materials such as methylene blue (MB) have been widely studied for PDT, the photothermal effect is rarely reported. Herein, mono-component nanoparticles lactic-*co*-glycolic acid-coated methylene blue (MBNPs) based on methylene blue (MB) and lactic-*co*-glycolic acid (PLGA) were prepared by a facile solution-based emulsification method at room temperature. The resulting nanoparticles possess high photothermal conversion efficiency and excellent photodynamic effect. For the first time, the *in vitro* and *in vivo* tests indicated an enhanced antitumor efficacy for MBNPs with combined PDT and PTT. This study provides an efficient approach to fabricate nano-MB and also demonstrates the great potential of lactic-*co*-glycolic acid-coated MB for biomedical applications. Most importantly, the strong tumor growth inhibition by synergistic PTT and PDT demonstrates an excellent cascaded synergistic effect of MBNPs for the tumor therapy.

## Abstract 2

Photodynamic therapy (PDT) and photothermal therapy (PTT) are synergetic treatment strategies in antitumor treatment to achieve the best anticancer efficacy.

## Graphical abstract

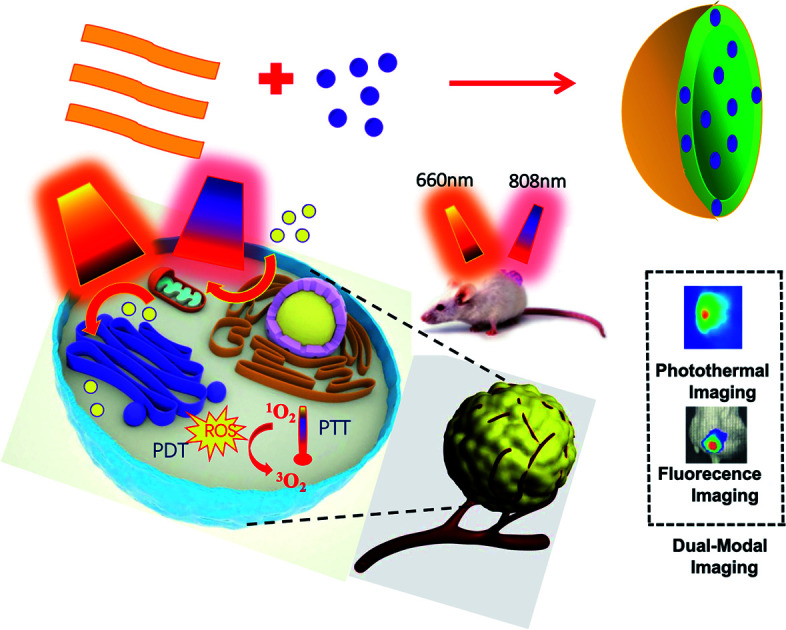

## Introduction

With the increasing number of tumor cases year by year, cancer treatment is one of the most difficult problems in human medicine in this century. Phototherapy, typically implemented in the forms of photothermal therapy (PTT) and photodynamic therapy (PDT), is a promising strategy for cancer therapy owing to its non-invasiveness and high selectivity.^1^ Recently, it has not only been demonstrated that synergistic PTT and PDT of phototherapy has shown enhanced anticancer efficiency compared with sole PTT or PDT, but also two-photon excitation with precise manipulation of treatment dose is more preferable than one-photon excitation, which may cause potential photo-damage to the adjacent tissues around tumor sites.^2^ Shao jun Guo *et al.* constructed a black phosphorus (BP)-based drug delivery system, which can achieve pH-/photo-responsive drug release, ^1^O_2_ generation with a 660 nm laser, and photothermal activity with an 808 nm laser.^3^ Hongjie Zhang *et al.* reported biocompatible copper ferrite nanospheres with enhanced reactive oxygen species (ROS) production under irradiation with a 650 nm laser through direct electron transfer and photo-enhanced Fenton reaction, and high photothermal conversion efficiency upon exposure to an 808 nm laser.^4^ Therefore, we chose dual light sources in the experiment.

PDT induces tumor cell death by transferring the energy of the photoexcited photosensitizer (PS) to oxygen to produce highly toxic ROS,^5^ such as singlet oxygen (^1^O_2_), superoxide anion 
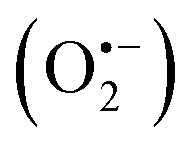
 and hydroxyl radicals (˙OH), which only takes place when the PS is irradiated with light.^2^ The PS drugs themselves are minimally toxic without the presence of external photo-activating light.^6^ The hydrophilic molecule MB, as a second generation PS, afforded increased tissue penetration depth and high biomedical imaging resolution.^7,8^ It is FDA-approved to be used in humans in the treatment of methemoglobinemia and has been used as a promising photosensitizer for PDT in the treatment of viruses, bacteria and cancer cells.^9–11^ All these properties make it a good photosensitizer for use in photodynamic therapies. MB is an inexpensive dye that exhibits a wide light absorption window (600–900 nm), with a peak at 660 nm, presenting low dark toxicity.^12^ This PS has exhibited great antitumor capabilities under the exposure to 660 nm and 808 nm lasers. However, there were few studies on the photothermal properties of MB nanoparticles, such as PPy–MB NPs^13^ and GBPs@SiO_2_-MB^14^ nanoplatforms synthesized in 2017, MDA-MB-468 investigated by V. P. S. Jesus *et al.* in 2018,^15^ and GO-MB/PF127, in which the photothermal properties of only GO-MB/PF127 and PPy–MB NPs were reported. Also, MB has the drawbacks of poor photo-stability and enzymatic degradation^16^ due to the aggregation-caused quenching (ACQ) effect.

In this study, we introduced the facile and economic synthesis of MB encapsulated PLGA nanoparticles to fabricate a novel NIR photo-absorber for a combination of PTT and PDT to overcome its limitations. As a nano- and microparticle prepared from biodegradable polymers, PLGA has been studied extensively for the sustained delivery of therapeutic agents including DNA, proteins and low molecular weight pharmacological agents.^17,18^ The unique structure of PLGA NPs, composed of a hydrophilic surface and a hydrophobic core, provides a carrying reservoir and also enables them to dissolve in aqueous solutions.^19^ One of the major advantages of encapsulation would also be the protection of the antigen or the drug from premature release and degradation.^17^ The singlet oxygen produced by MBNPs will exist longer because the PLGA molecules affect the aggregation state of MB, reducing the ACQ effect. Another advantage is that the by-products of PLGA, the lactic acid and glycolic acid, can be eliminated from the body as carbon dioxide and water through the tricarboxylic acid cycle.^19^ In summary, PLGA showed promise for biomedical applications, owing to its excellent biocompatibility, low cytotoxicity and metabolism in living tissues.^19^

The ability to visualize anatomical structures in a living biological system has revolutionized the treatment efficacy in day-to-day clinical practice. Photoacoustic imaging (PAI) combining both optical and acoustic features offers advantages which are nearly impossible to achieve by using any of the pre-existing conventional imaging tools at the depth beyond the limit of optical detection.^20^ Moreover, MBNPs theoretically possess excellent PAI capability because of high NIR absorption per unit mass or per molar concentration,^20^ which is useful for determining the tumor area before the treatment. Finally, the hyperthermia was characterized by the temperature change *via* a thermal imaging system and *in vitro* cytotoxicity and *in vivo* anticancer efficiency were examined to demonstrate the superiority of this PAI-guided cascaded synergistic effect.

**Scheme 1:**
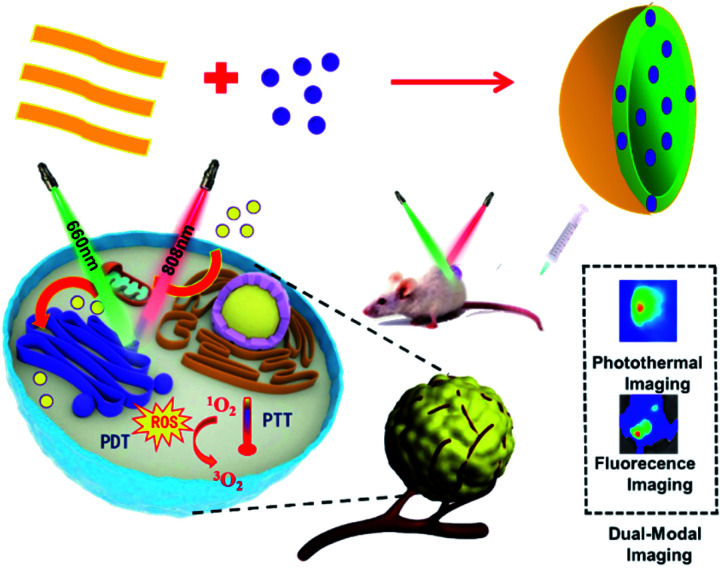
Preparation and application of MBNPs for PDT and PTT.

So far, there have been no reports about the utilization of porphyrin-containing two-dimensional conjugated polymers for combined PDT and PTT. Therefore, in this study, mono-component nanoparticles lactic-*co*-glycolic acid-coated methylene blue (MBNPs) based on MB and PLGA were synthesized by a facile solution-based emulsification method at room temperature, and then for the first time, the antitumor efficacy *via* synergetic PDT and PTT was evaluated *in vitro* and *in vivo*, as depicted in Scheme 1.

## Results and discussion

### Synthesis and characterization of MBNPs

**Fig. 1:**
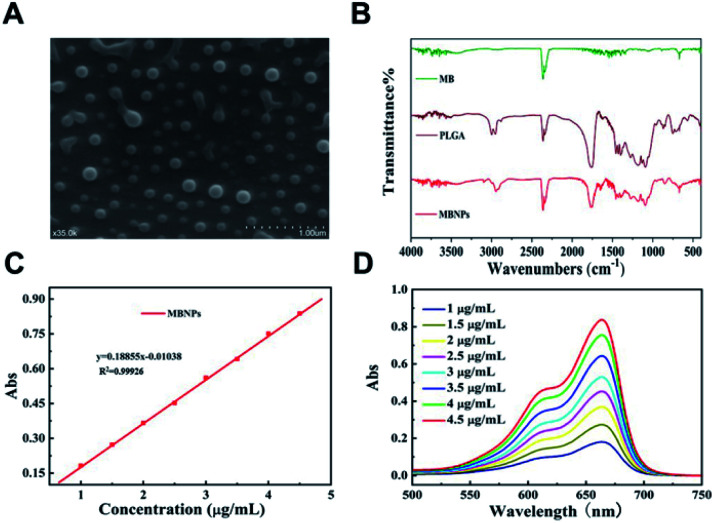
(A) MBNP SEM images. (B) FTIR spectra of toluidine blue (MB), lactic-*co*-glycolic acid (PLGA), and PLGA-coated MB nanoparticles (MBNPs). (C) Standard curve line of MB (663.5 nm). (D) Ultraviolet-visible absorption spectra of MB.

The morphology of the prepared MBNPs was characterized by scanning electron microscopy (SEM) and Fourier-transform infrared spectroscopy (FTIR). Fig. 1A shows the characterization of MBNPs by SEM, from which we can find that the size of MBNPs was ranging from 200 to 300 nm, as a uniform appearance. The results proved that polylactic acid successfully coated MB. The dynamic light scattering (DLS) spectra showed that ultrafine MBNPs with an average hydrodynamic diameter of 244.8 nm and a polydispersity index (PDI) of 0.153 were formed (Fig. S1 and S2†). TBNPs are negatively charged, and zeta potential was −0.122 mV (Fig. S3†). The drug encapsulation efficiency and loading capacity of MB were calculated to be 2.4 wt% and 81.5% respectively. The FTIR spectra of PLGA and MBNPs showed the absorption peaks around 3000 cm^−1^ and at 1000 to 1750 cm^−1^, as there was no absorption in MB in the same regions (Fig. 1B). In general the absorption peaks of PLGA were deeper than those of MBNPs; by inference, MB was successfully coated with PLGA due to coherent cancellation. The absorption band at 2300 cm^−1^ originated from the stretching mode of the CN chains. The absorption band at 1750 cm^−1^ belonged to the bending vibration of the benzene ring C–C skeleton of MB, and the absorption band at 700 cm^−1^ belonged to the Cl-1 absorption peak. Through ultraviolet-visible absorption spectroscopy, it was found that MBNPs exhibited a wide range of absorption from ultraviolet to near infrared at different concentrations (1, 1.5, 2, 2.5, 3, 3.5, 4 and 4.5 μg mL^−1^) (Fig. 1C and D). Further, the maximum absorbance at 663.5 nm is the characteristic wavelength for MBNPs, as MBNPs displayed strong photoluminescence (PL) emission (Fig. S4 and S5†).

### Photodynamic effect of MBNPs

**Fig. 2:**
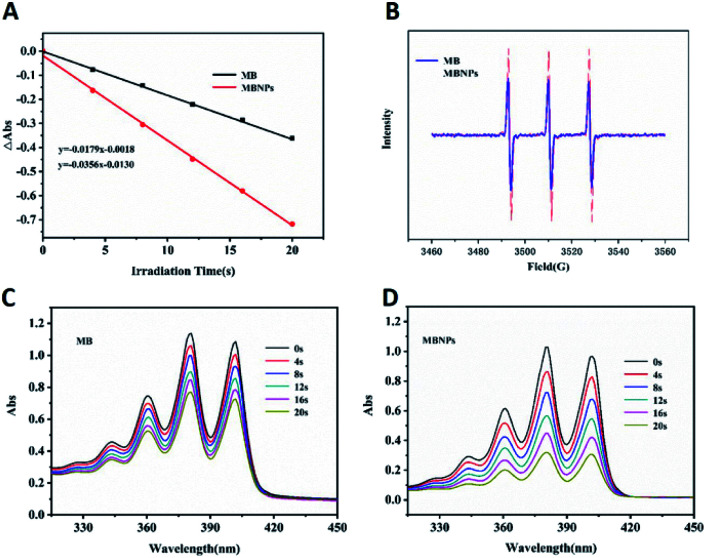
(A) ABDA absorption curve of MB and MBNPs in water. (B) EPR spectrum of MB and MBNPs. Absorption spectra of ABDA mixed with (C) MB and (D) MBNPs in aqueous solution with 660 nm laser irradiation.

The efficiency of ^1^O_2_ generation was used to access the photodynamic activity of MB and MBNPs by a probe 9,10-anthracene di-bis(methylene)dimalonic acid (ABDA). The results showed that the slope of the decay curves of MB and MBNP absorption is observed to be proportional to the efficiency of ^1^O_2_ generation in water (Fig. 2A). Compared with the MB curve, the MBNP sample showed a steeper decline within 20 min under the radiation of 660 nm, indicating a better capability of ^1^O_2_ generation, and the capability was time-dependent. MBNPs showed better photodynamic stability than MB by electron paramagnetic resonance (EPR) analysis (Fig. 2B). These results were also confirmed by electron spin resonance (ESR) using 2,2,6,6-tetramethylpiperidine (TEMP) as an ^1^O_2_ scavenger (Fig. 2C and D).

### PTT experiment of the MBNPs *in vitro*

**Fig. 3:**
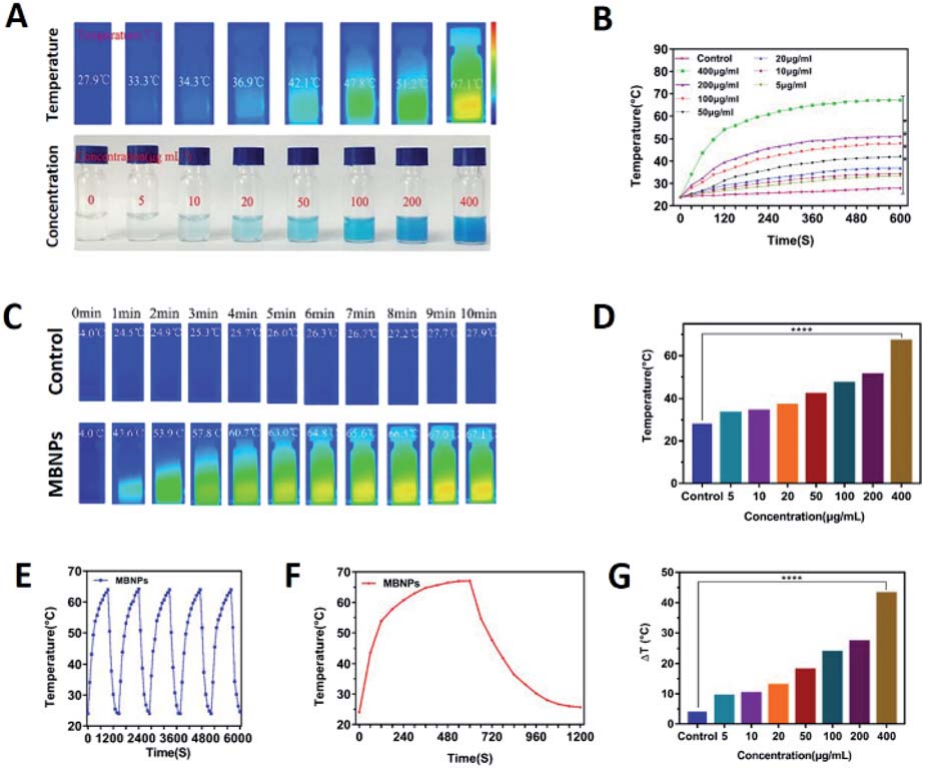
(A) Optical photographs and thermal images of MBNPs at different concentrations (0, 5 μg mL^−1^, 10 μg mL^−1^, 20 μg mL^−1^, 50 μg mL^−1^, 100 μg mL^−1^, 200 μg mL^−1^, and 400 μg mL^−1^) in PBS solutions after laser irradiation. (B) Photothermal images of MBNPs and water under 808 nm laser irradiation (1.0 W cm^−2^, 10 min). (C) Temperature change of MPNPs (400 μg mL^−1^) irradiated with an 808 nm laser; the laser was turned off after 300 s. (D) Temperature change curves of MPNPs at different concentrations (0–400 μg mL^−1^, 1 mL) under laser irradiation (808 nm, 1.0 W cm^−2^) as a function of the irradiation time. (E) The temperature variations of MBNPs at 400 μg mL^−1^ under 808 nm laser irradiation for 5 cycles. (F) Temperature change curves of MPNPs at different concentrations (0–400 μg mL^−1^, 1 mL) under laser irradiation (808 nm, 1.0 W cm^−2^) as a function of the irradiation time. (G) Temperature change histogram of water and MBNP solution with different concentrations (control, 5, 10, 20, 50, 100, 200 and 400 mg mL^−1^) under the same heating conditions.

The photo-sensitivity of the MBNPs is first confirmed by irradiation with an 808 nm laser with an intensity of 1.0 W cm^−2^. The correlation of temperature rise with concentration and time was verified. Fig. 3C and F showed that the temperature of MBNP aqueous suspension at 400 μg mL^−1^ increases rapidly by ∼67.1 °C within 10 min of irradiation, whereas the temperature of the group treated with pure water showed little elevation (*T*_max_ = 27.9 °C). The temperature of MBNPs decreased rapidly as soon as we stopped giving irradiation. The photothermal conversion performance of the MBNPs remained excellent and stable after five cycles of laser on/off (Fig. 3E). These findings suggest that MBNPs have long-term photothermal stability. Moreover, Fig. 3A and D showed that the temperature change in MBNP solution was concentration dependent. At the same irradiation time, using the data at 10 min as the example, the solution temperature is increasing along with the increase in concentration of MBNPs, and 0.4 mg mL^−1^ MBNP solution could even reach up to 67.1 °C. The temperature increase (Δ*T* ≈ 43.1 °C) of MBNPs was much higher than that of water (Δ*T* ≈ 3.9 °C) after laser irradiation (808 nm, 1.0 W cm^−2^, 10 min). Fig. 3G shows the change of temperature rising (Δ*T*) with the concentration directly. Fig. 2B is a summary of the variation of MBNP heating efficiency with time and concentration. These results showed that MBNPs have excellent photodynamic and photothermal properties.

### PDT experiment of the MBNPs in cells

**Fig. 4:**
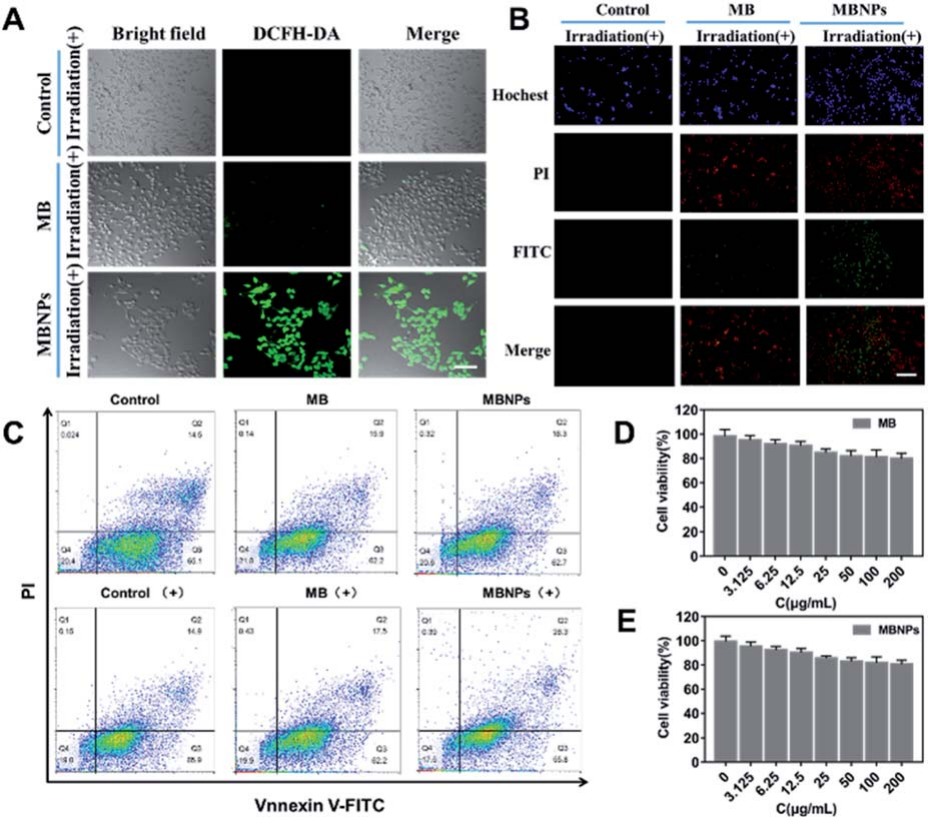
(A) Confocal laser scanning microscope (CLSM) images of DCFH-DA stained 4T1 cells incubated with MB and MBNPs before and after irradiation with a 660 nm laser (50 mW cm^−2^, 5 min). (B) Confocal laser scanning microscope images of 4T1 cells co-stained by Annexin V-FITC and PI at different incubated times after irradiation with a 660 nm laser. (C) Confocal FL images of DCFH-DA stained 4T1 cells incubated with MB and MBNPs before and after irradiation with a 660 nm laser (50 mW cm^−2^, 5 min). Activities of 4T1 cells incubated with MB (D) and with MBNPs (E) at different concentrations (0, 3.125, 6.25, 12.5 25, 50, 100, and 200 μg mL^−1^).

The 2,7-dichlorofluorescein diacetate (DCFH-DA) dye can be oxidized to DCF by the presence of ^1^O_2_ in cells, showing green fluorescence when excited at 520 nm. Therefore, DCFH-DA, as a ROS-index probe, was used to determine the generation of ^1^O_2_ with incubation of MBNPs and MB under NIR irradiation. As shown in confocal laser scanning microscope (CLSM) images of 4T1 cells, the control groups without any treatment showed no fluorescence, and only very weak green fluorescence was observed in the group with MB occasionally, whereas bright green fluorescence can be seen in MBNPs, implying that the MBNPs induce the production of ^1^O_2_ under NIR irradiation more efficiently than MB and can be used as an alternative dynamic agent (Fig. 4A). These results demonstrate that MBNPs can be used for efficiently treating cancer cells by PDT due to the excellent photo-sensitivity and the production of hydroxyl radicals.

The anticancer efficacy of MBNPs and MB both with dual light irradiation was evaluated *in vitro* using Annexin V-FITC and PI double staining (Fig. 4B). The 4T1 cells were incubated for 12 h with MB and MBNPs respectively. There were obviously more cells tinting with green fluorescence in the MBNP group compared with control and MB groups. The results showed that there were more cells at the early stage of apoptosis in the group treated with MBNPs than MB under irradiation. But the red FL signals did not change much compared with the control group which indicated that the cells were at the late-stage apoptotic state in both MBNP and MB groups. Apoptotic cells were quantitated by the Annexin V-FITC/PI double staining method in flow cytometry (Fig. 4C). The apoptosis rate of 4T1 apparently rose in MBNPs + irradiation as 27.6% compared with the other groups, especially compared with MB + irradiation and MBNP groups. By the way, the cytotoxicities of the MBNPs and MB were also evaluated in 4T1 cells by MTT assay. The viability of cells is more than 80% at 200 μg mL^−1^ respectively (Fig. 4D and E). Based on the above experimental results, we can conjecture that MBNPs have no obvious toxicity to cells in the absence of light activation, and have better photothermal performance than MB.

### *In vivo* therapeutic effect on tumor

**Fig. 5:**
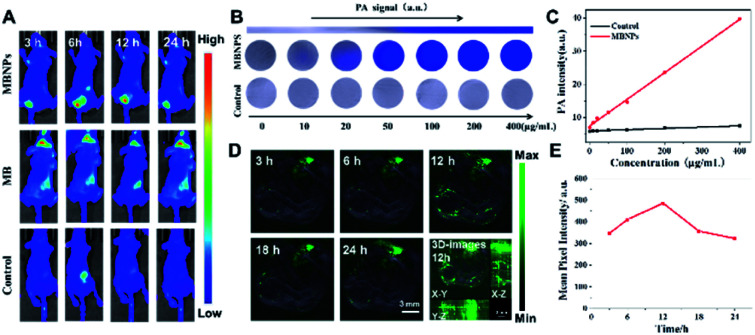
(A) Representative fluorescence images taken at different time points after injection of MB and MBNPs. (B) *In vitro* PA imaging of the MBNPs and control solution at different concentrations. (C) The corresponding quantitative curve of PA intensity at different concentrations. (D) PA images of 4T1 tumor-bearing mice after i.v. injection of MBNPs (400 μg mL^−1^, 100 μL). (E) The corresponding PA-signal of MBNPs at different time points.

Based on the results above, we further explored the synergistic therapeutic effect of MBNPs *in vivo* with mice. Animal experimentation was governed by the Regulations of Experimental Animals of Beijing Authority. The Animal Ethics Committee of the China Agriculture University approved the experiment. The photoacoustic (PA) spectrum of MBNPs in PBS solution is measured in the spectral region of 600–900 nm, with the maximal intensity at about 680 nm. We further recorded the PA images and the corresponding PA intensities of the MBNPs and control; it has been illustrated that the MBNP component could serve as a contrast agent for PAI. Therefore, the possibility of MBNPs for PAI was firstly assessed *in vitro*. As depicted in Fig. 5A and B, the PA amplitudes of the MBNPs at 680 nm were determined in a series of concentrations from 0 to 400 μg mL^−1^ based on MBNPs, showing a relatively good linear relationship in a large range. As shown in Fig. 5B, in comparison with the PBS, the MBNPs exhibit much better PA conversion performance, which is in accordance with the photothermal conversion property, and would enable a superior PAI effect.

Based on the PA effect of MBNPs, the accumulation of MBNPs can be tracked in tumor tissue by PA imaging to further determine the tumor position and optimize the treatment time. The PA cross-sectional scan images of 4T1 tumor-bearing mice were obtained at different times after intravenous (i.v.) injection with MBNPs (400 μg mL^−1^, 100 μL). The results showed that the accumulation of MBNPs in the tumor region gradually increased and reached the maximum at 12 h, indicating that MBNPs could passively target the tumor region through the EPR effect (Fig. 5C and D).

**Fig. 6:**
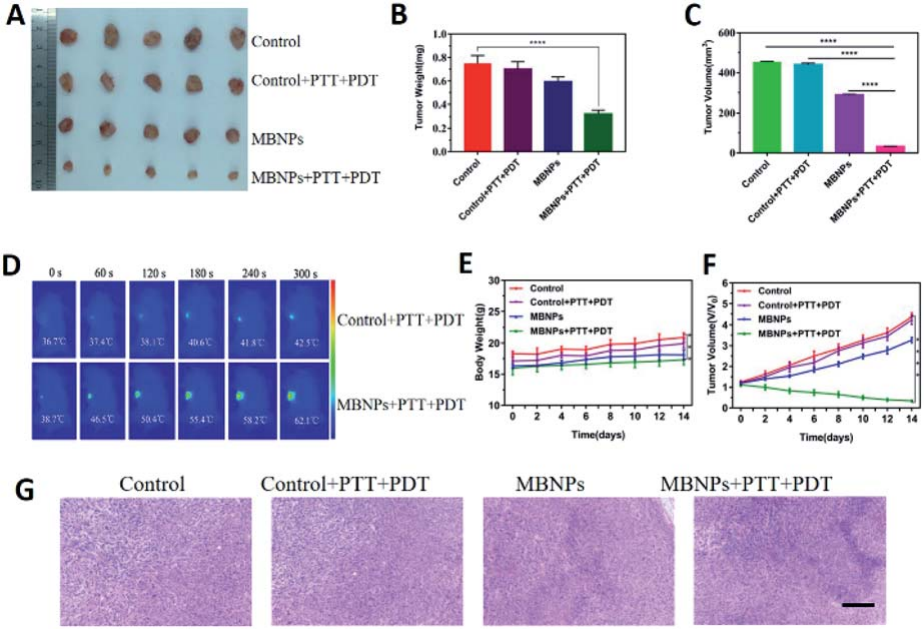
(A) Photographs of dissected tumors of different groups (control, control + PTT + PDT, MBNPs, and MBNPs + PTT + PDT). (B) Average weights of tumors at day 14 after treatment. (C) Average volumes of tumors at day 14 after treatment. (D) *In vivo* photothermal images of the corresponding temperature change in mice after the injection of PBS and MBNPs under 808 nm laser irradiation (1 W cm^−2^, 5 min). (E) Body weight of mice treated with different methods. (F) The relative tumor volume of mice treated with different methods. (G) The H&E staining in the tumor site in each group with different treatments (scale bar, 100 μm).

The tumor suppressive ability of MBNPs is studied by dividing the mice bearing 4T1 breast tumors into four groups randomly (5 mice per group): (1) control; (2) control + PTT + PDT; (3) MBNPs; (4) MBNPs + PTT + PDT. The MBNPs were injected intravenously (i.v.) (400 μg mL^−1^, 100 μL). The laser irradiation (combined 808 nm and 660 nm, 1 W cm^−2^, 5 min) was administered at 12 h post-injection, and the tumor volumes and tumor weights in each group were recorded (Fig. 6A). As shown in the representative digital photos of tumor-bearing mice before and after different therapies in Fig. S10,† the tumor in the MBNP + PTT + PDT group is really small, which indicates the significant therapeutic effect of MBNPs + PTT + PDT. The relative tumor volumes and tumor weights of all the groups are illustrated in Fig. 6B and C to confirm the cancer therapeutic effect. The tumor volume and weight results revealed that MBNPs + PTT + PDT exhibited the better therapeutic efficiency compared with the other groups. Additionally, the weights of tumor bearing mice which were in the MBNP + PTT + PDT group were only mildly decreased compared with other groups maintained after prolonged therapy (Fig. 5F). We can consider that the MBNP theranostic agent showed good biocompatibility and safety.

Motivated by the attractive tumor accumulation of MBNPs, their antitumor potency was investigated using laser intensity (1.0 W cm^−2^) irradiation. *In vivo* photothermal images showed that a marginal temperature increase of only Δ*T* ≈ 5.8 °C was observed from the Laser group (saline + laser) (Fig. 6D and E). On the contrary, tumors in the MBNP + Laser group exhibited a rapid temperature increase of ≈23.4 °C, suggesting that the MBNPs demonstrated an effective *in vivo* photothermal conversion.

The histologic analysis of tumors was also performed to assess the tumor treatment effect besides direct data above. The tumors of all the groups were removed after 14 days of treatment, which were ready for H&E histologic analysis (Fig. 6H). The tumor cells were obvious and grew quickly, and no sign of necrosis was observed in the control and control + PTT + PDT groups. Extensive necrosis area in the tumor site of the MBNP + PTT + PDT group was apparent such as the dense and unclear structure, excessively dyed by eosin, compared with MBNPs groups for which mild necrosis area can be observed. The results of H&E histologic analysis were in agreement with the tumor suppressive effect of *in vivo* PTT + PDT performance of MBNPs under 660 nm and 808 nm lasers.

### Study of MBNP bio-safety

**Fig. 7:**
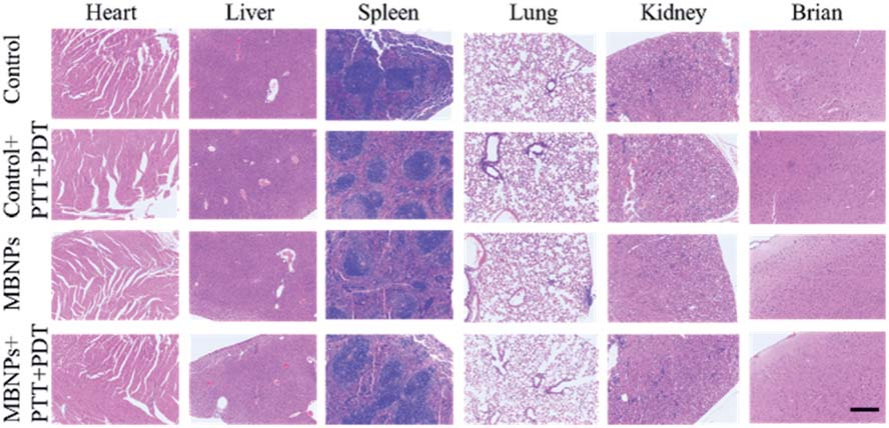
Photomicrographs of H&E stained slices of the major organs from the mice 7 days after intravenous injection with saline (100 μL), MB (200 μg mL^−1^, 100 μL), and MBMPs (200 μg mL^−1^, 100 μL). The scale bar is 500 μm.

The biological safety of MBNPs was also verified in mice through the hemolysis test and histologic analysis. The results of the hemolysis test showed that no significant hemolysis occurred with the concentration of the MBNP solution up to 400 μg mL^−1^ (Fig. S6†). The hemolysis rate was less than 5% and the red blood cell morphology was normal (Fig. S7 and S8†). Fig. S9† shows that after 24 h of incubation with MBNPs, there is no observable change to the relative viability of 4T1 cells even at the concentration of up to 200 μg mL^−1^, indicating low cytotoxicity of the tested nanomaterials. The histologic analysis in the tissues (heart, liver, spleen, lungs, kidneys and brain) through the H&E staining indicated no noticeable damage or inflammation from all groups (Fig. 7). These results showed good biocompatibility of MBNPs.

## Conclusions

In summary, biocompatible and biodegradable MBNPs were demonstrated for the obvious efficient treatment of cancer through the synergy of thermal therapy and dynamic therapy under dual NIR (808 nm/660 nm) irradiation through the enhanced generation capability of the ^1^O_2_ and ROS in tissues. The MBNPs show synergistic PDT/PTT with excellent efficiency for killing tumor cells *in vitro* and inhibiting tumor growth *in vivo* at very low doses by dual NIR irradiation. The PLGA nanoplatform represents excellent ultrahigh drug-loading capacity of MB and good biocompatibility. Further clinical applications of MBNPs with dual NIR irradiation could be considered.

## Experimental

### Preparation of MBNPs

MB (Sigma-Aldrich, USA) solution (10 mg mL^−1^) with 3% NaCl was used as the internal aqueous phase. 1% and 0.5% PVA (Jinan Daigang Biological Engineering Co. Ltd) solution was configured as the external aqueous phase. 200 mg PLGA (Jinan Dai gang Biological Engineering Co. Ltd) was weighed and dissolved in 5.5 mL ethyl acetate. 0.5 mL the inner water phase was put into the oil phase, and the colostrum is formed by ultrasound under an ice bath. The colostrum was poured into 10 mL of 1% PVA external water phase, and the pre-mixed milk was prepared with an ultrasonic ice bath. The premixed milk was poured into 10 mL 0.5% PVA solution to produce the premixed milk, and stirred at 300–400 rpm at room temperature for 3–4 h and PLGA nanoparticles were obtained. Then, the solution was centrifuged at 4 °C at 10 000 rpm for 10 min and distilled water was added for ultrasonic dispersion.

### Cell culture and treatment

4T1 cells (mouse mammary tumor cell line) were maintained in high-glucose Dulbecco's Modified Eagle's Medium (DMEM), which was supplemented with 10% fetal bovine serum, 100 units per mL penicillin, and 100 μg mL^−1^ streptomycin at 37 °C under a humidified atmosphere of 5% CO_2_. The cytotoxicity of MBNPs was evaluated by 3-(4,5-dimethylthiazol-2-yl)-2,5-diphenyltetrazolium bromide (MTT) viability assay. After co-incubating the cells with MPNPs for 24 h at a series dosage, MTT solution was added to each well. After 4 h of incubation at 37 °C, colorimetric measurements were performed at 495 nm on a scanning multi-well spectrometer. Data were expressed as mean ± standard error of mean of at least five independent experiments.

### Detection of singlet oxygen

SMBNPs and MB were dispersed in water solution for single oxygen tests. The detailed procedure was based on the method described previously. The trapping agent utilized in this test was DMPO. The trapping agent was pipetted into solution and mixed homogeneously as preparation and then capillaries were introduced to load samples. Then the capillaries were placed into an Electron Paramagnetic Resonance (JEOL, JES-FA200) to collect information of radicals.

### Intracellular ROS detection

4T1 cells were incubated with different concentrations of MBNPs first, and then the culture medium was replaced and washed three times. DMEM containing DCFH-DA (10 × 10^−6^ M) was added and further incubated for 40 min, and subsequently, it was irradiated with a 660 nm laser for 10 min (50 mW cm^−2^). The results were characterized by fluorescence micrography. The nuclei of 4T1 cells were stained with 4,6-diamidino-2-phenylindole (DAPI).

### *In vitro* cell viability, PDT and PTT treatment against 4T1 cells

Four groups of HeLa cells harvested in a logarithmic growth phase were seeded in a 96-well plate with a density of 7 × 10^3^ cells per well, and incubated in DMEM containing 10% fetal bovine serum for 24 h. The media were replaced by culture medium containing MBNPs with different concentrations (3.125, 6.25, 12.5, 25, 50, 100, 200 μg mL^−1^, and 100 μL). After 4 h, three of the four groups were irradiated with a 660 nm laser (200 mW cm^−2^, 10 min), an 808 nm laser (1.2 W cm^−2^, 5 min), and a 650 nm laser (200 mW cm^−2^, 10 min) as well as an 808 nm laser (1.2 W cm^−2^, 5 min), respectively. The cells were incubated for another 24 h. 20 μL of MTT solution with a concentration of 5 mg mL^−1^ was added and the plates were incubated for another 4 h at 37 °C, followed by removal of the culture medium containing MTT and addition of 150 μL of dimethyl sulfoxide (DMSO) to each well to dissolve the formed formazan crystals. Finally, the plates were shaken for 5 min, and the absorbance of the formazan product was measured at 490 nm with a microplate reader.

### *In vivo* antitumor efficacy of MBNPs

Experimentation with animals was governed by the Regulations of Experimental Animals of Beijing Authority and approved by the Animal Ethics Committee of the Peiking University. Female BALB/C mice (provided by Vital River Laboratory Animal Technology Co. Ltd., Beijing), aged 6–8 weeks, were used in the experiments. Mice were raised in independent ventilated cages and received pathogen-free food and water, and the animal experiments agreed with the criterions of The National Regulation of China for Care and Use of Laboratory Animals. The tumors were established by subcutaneous injection of 4T1 (murine hepatocarcinoma cell lines) cancer cells in the left axilla of the mice. The tumor bearing mice were randomly divided into four groups (*n* = 5, each group) after the size of the tumors reached 100–120 mm^3^, and treated with PBS, PBS + 660 nm + 808 nm (PBS + PDT + PTT), MBNPs, and MBNPs + 660 nm + 808 nm (MBNPs + PDT + PTT), respectively. 100 μL of MBNPs with a concentration of 1 mg mL^−1^ was intra-tumorally injected into mice for the *in vivo* experiments, and the mice were treated two times on day 1 and day 7, respectively, at each time accompanied by the NIR laser irradiation (650 nm for 5 min, 500 mW cm^−2^; 808 nm for 5 min, 500 mW cm^−2^). The body weight and tumor volume of each mouse were monitored every two days, and after 14 days of treatment, the tumors were dissected and weighed to evaluate the therapeutic efficacy. In a typical calculation, the tumor volume was calculated by volume = length × (width)2/2, and the relative tumor volume was calculated as *V*/*V*_0_, where *V*_0_ is the tumor volume before the treatment. Finally, the major organs of mice, such as the liver, spleen, heart, lungs, and kidneys, were removed and fixed in 4% paraformaldehyde solution for histological examination in order to further investigate the biocompatibility of MBNPs *in vivo*.

### Statistical analyses

Statistical analysis was performed with SPSS (Statistical Product and Service Solutions). All experimental data are presented as mean ± SD. For comparison of multiple experimental groups, two-way ANOVA was used. A *p* value < 0.05 was considered statistically significant: *p < 0.05 and **p < 0.01 indicate significant differences.

## Conflicts of interest

There are no conflicts to declare.

## Supplementary Material

RA-012-D1RA07689B-s001
