# Supplementary material for: PLGA-coated methylene blue nanoparticles for photoacoustic imaging and photodynamic/photothermal cascaded precisely synergistic therapy of tumor
Source: RSC Adv. 2022 Jan 10;12(3):1543–9. doi: 10.1039/d1ra07689b (PMC8978873; doi:10.1039/d1ra07689b)
Supplement: RA-012-D1RA07689B-s001 [file RA-012-D1RA07689B-s001.pdf]

Electronic Supplementary Material (ESI) for RSC Advances.
This journal is © The Royal Society of Chemistry 2021

Supporting Information

PLGA-coated Methylene Blue Nanoparticles for Photoacoustic Imaging and Photodynamic/Photothermal Cascaded Precisely Synergistic Therapy of Tumor

Xiaomu Xu, †[12] Haiyan Wu, †[1] Yue Yang,[1] Bo Liu,[1] Jijing Tian,[1] Huihui Bao,[3] Tianlong Liu*[1]

1, College of Veterinary Medicine, China Agricultural University, No.2 West Road Yuanmingyuan, Beijing 100193, P.R. China.

2, College of Chemistry and Materials Science, Jinan University, No. 601, West Huangpu Avenue, Guangzhou, Guangdong 510632, China.

3, China National center for food safety risk assessment, No.37, Guangqu Road, Chaoyang District, Beijing 100022, P.R. China.

† These authors contributed equally.

Correspondence: Tianlong Liu

Email: liutianlong@cau.edu.cn

Supporting Information

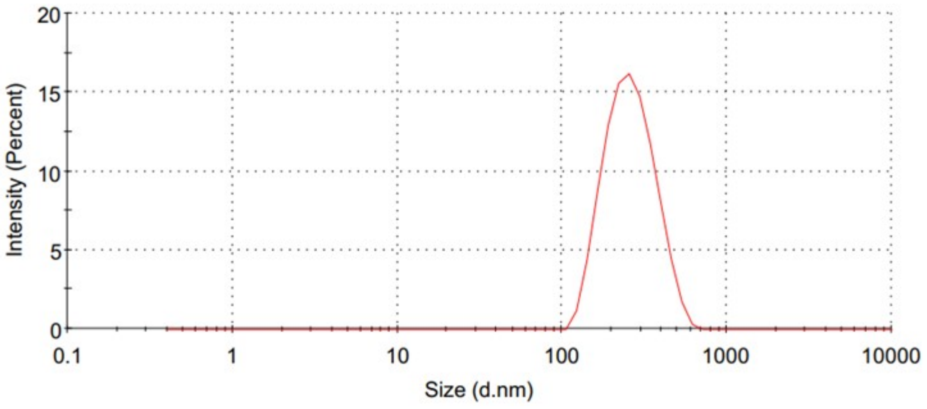

Figure S1. The DLS analysis of MBNPs in water.

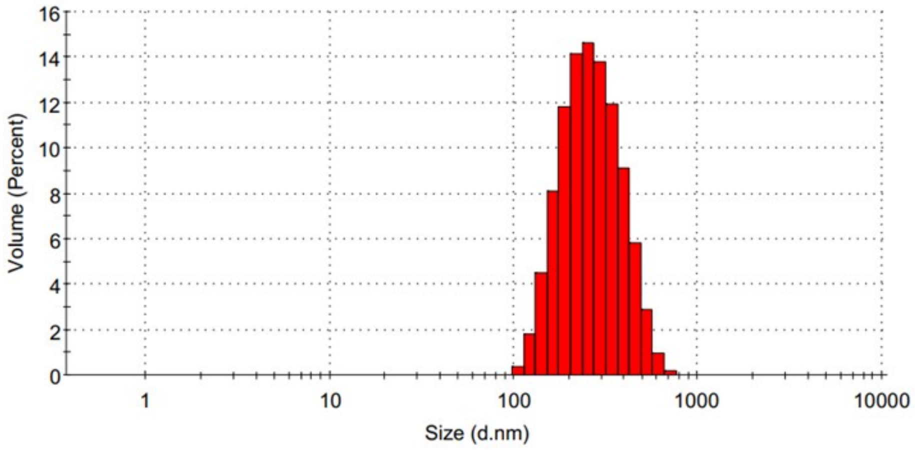

Figure S2. Hydration diameter distribution of MBNPs in water.

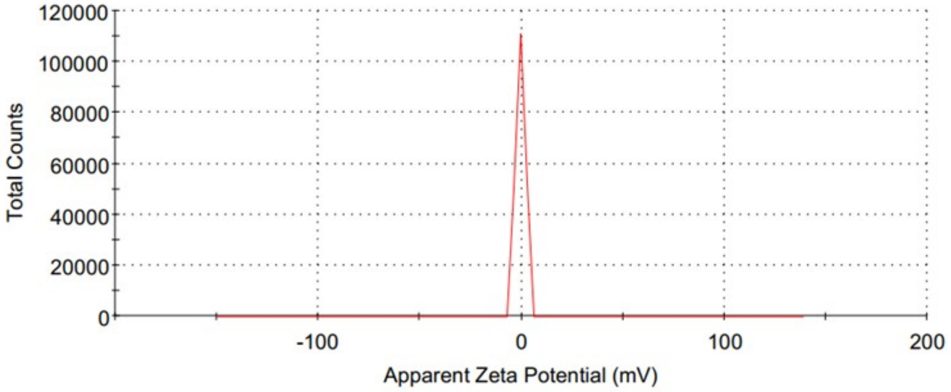

Figure S3. Zeta of MBNPs in pure water.

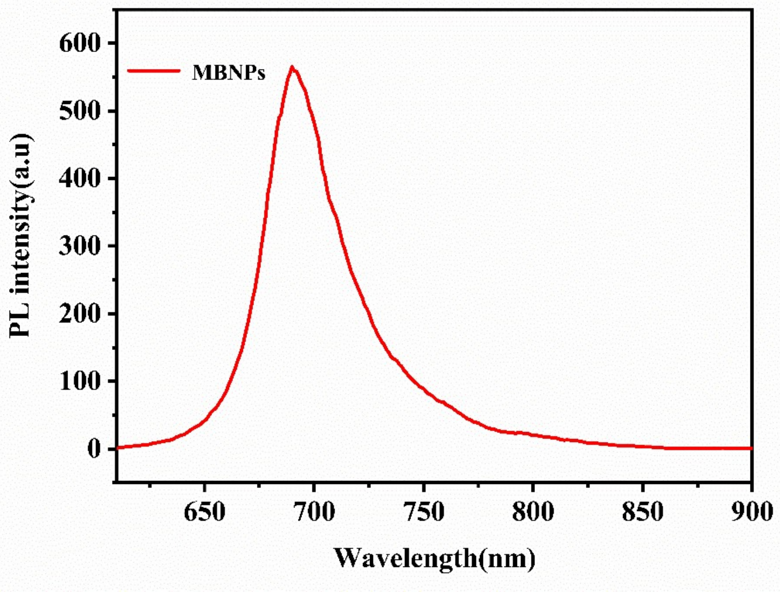

Figure S4. UV–Vis spectrum of MBNPs.

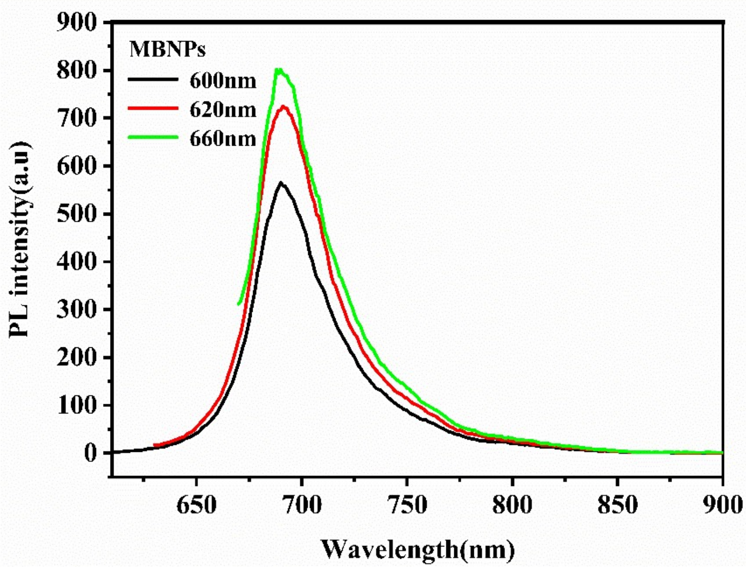

Figure S5. Normalized UV-Vis spectra of MBNPs in aqueous solution.

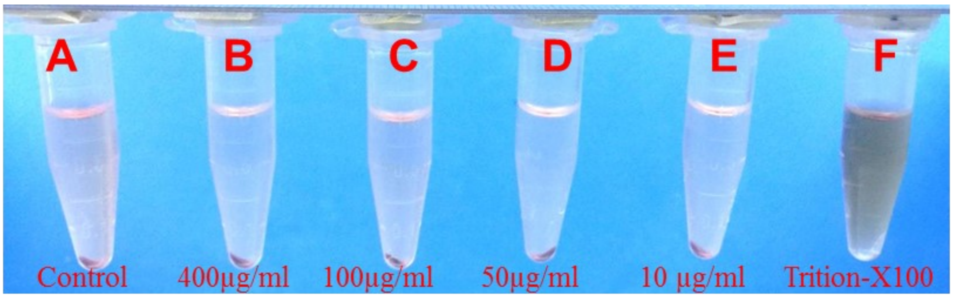

Figure S6. Hemolysis of red blood cells (RBCs) treated with control,

MBNPs (3.125, 6.25, 12.5, 25, 50, 100, and 200 μg mL−1) and Triton-X100 solution.

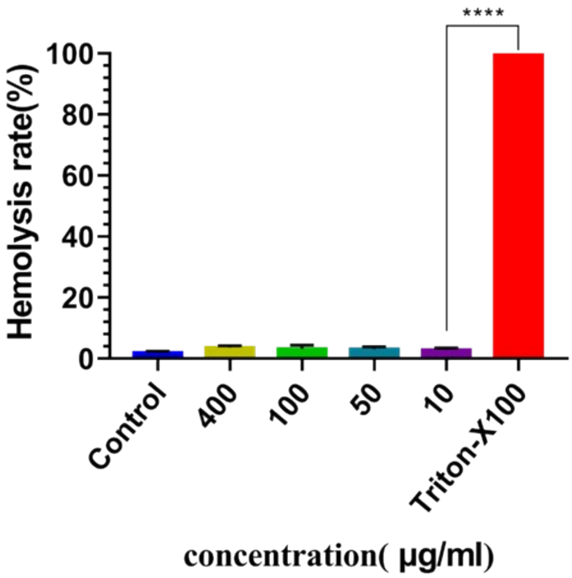

Figure S7. Hemolysis percentage of red blood cells (RBCs) treated with MBNPs solution.

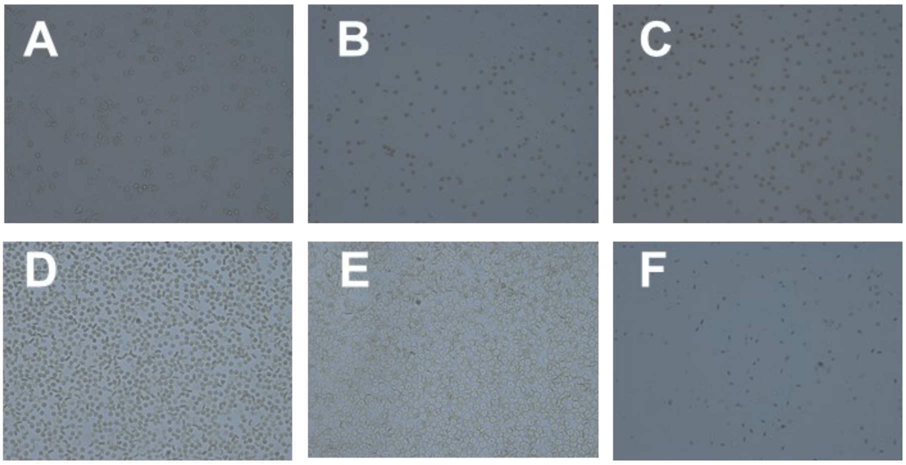

Figure S8. The photograph under a microscope of red blood cells (RBCs) .

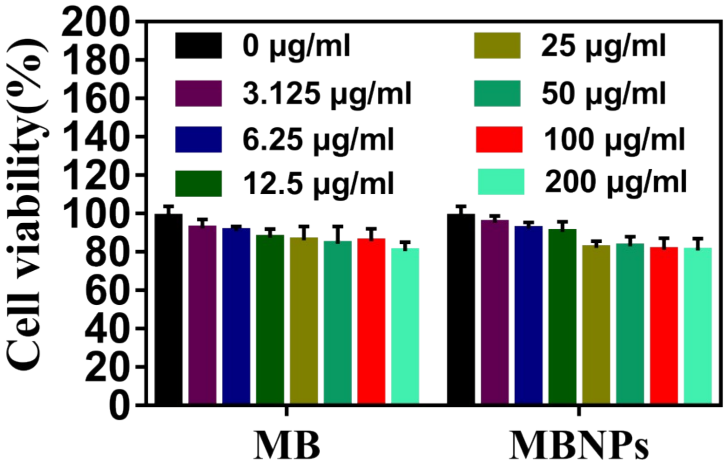

Figure S9. Relative cell viability of 4T1 cells after 24 h incubation with MBNPs (3.125, 6.25, 12.5, 25, 50, 100, and 200 μg mL^{-1}).

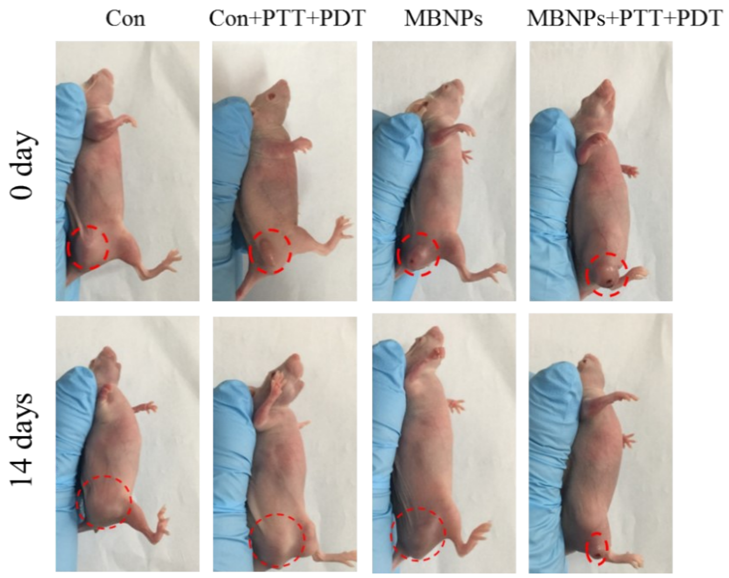

Figure S10. Representative digital photos of tumor-bearing mice before and after different therapies. The mice were treated with control group, injection of saline with 660 nm and 808 nm laser irradiation, injection of MBNPs without laser irradiation, injection of MBNPs with vitamin C (VC) irradiated by 660 nm and 808 nm laser. The relative tumor volume.
